# Small RNA Profiling in an HTLV-1-Infected Patient with Acute Adult T-Cell Leukemia-Lymphoma at Diagnosis and after Maintenance Therapy: A Case Study

**DOI:** 10.3390/ijms241310643

**Published:** 2023-06-26

**Authors:** Rodrigo Pessôa, Daniela Raguer Valadão de Souza, Youko Nukui, Juliana Pereira, Lorena Abreu Fernandes, Rosa Nascimento Marcusso, Augusto César Penalva de Oliveira, Jorge Casseb, Alberto José da Silva Duarte, Sabri Saeed Sanabani

**Affiliations:** 1 Postgraduate Program in Translational Medicine, Department of Medicine, Federal University of São Paulo (UNIFESP), São Paulo 04039-002, Brazil; rodrigo_pessoa1@hotmail.com (R.P.); daniraguer@gmail.com (D.R.V.d.S.); lorena.abreu.fernandes@gmail.com (L.A.F.); 2 Department of Hematology, Faculty of Medicine, University of São Paulo, São Paulo 05403-000, Brazil; youko.nukui@hc.fm.usp.br (Y.N.); juliana.pereira@hc.fm.usp.br (J.P.); 3 Department of Neurology, Emilio Ribas Institute of Infectious Diseases, São Paulo 01246-900, Brazil; rmmarcusso@gmail.com (R.N.M.); rdcassia@uol.com.br (A.C.P.d.O.); 4 Laboratory of Medical Investigation LIM-56, Division of Dermatology, Medical School, University of São Paulo, São Paulo 05403-000, Brazil; jcasseb@usp.br (J.C.); alberto.duarte@hc.fm.usp.br (A.J.d.S.D.); 5 Laboratory of Medical Investigation Unit 03, Clinics Hospital, Faculty of Medicine, University of São Paulo, São Paulo 05403-000, Brazil; 6 Laboratory of Dermatology and Immunodeficiency, LIM56/03, Instituto de Medicina Tropical de São Paulo Faculdade de Medicina da Universidade de São Paulo, Av. Dr. Eneas de Carvalho Aguiar, 470 3° andar, São Paulo 05403-000, Brazil

**Keywords:** small RNA, HTLV-1, ATLL, massive parallel sequencing

## Abstract

Small RNAs (sRNAs) are epigenetic regulators of essential biological processes associated with the development and progression of leukemias, including adult T-cell leukemia/lymphoma (ATLL) caused by human T-cell lymphotropic virus type 1 (HTLV-1), an oncogenic human retrovirus originally discovered in a patient with adult T-cell leukemia/lymphoma. Here, we describe the sRNA profile of a 30-year-old woman with ATLL at the time of diagnosis and after maintenance therapy with the aim of correlating expression levels with response to therapy.

## 1. Introduction

Human T-cell lymphotropic virus type 1 (HTLV-1) is an oncogenic human retrovirus that was originally discovered in a patient with adult T-cell leukemia/lymphoma (ATLL) [1]. It is estimated that 5–10 million people worldwide are infected with HTLV-1, but it is important to note that this number is still largely unknown in countries such as India, China, Russia, Australia, and several African countries [2]. HTLV-1 infection can cause a wide range of clinical symptoms, from asymptomatic infection to malignant ATLL and HTLV-1-associated myelopathy/tropical spastic paraparesis (HAM/TSP) [3]. Most HTLV-1 patients remain asymptomatic for life, but others advance to a preleukemic phase characterized by low numbers of circulating leukemic cells in peripheral blood and skin lesions but no involvement of other organ systems [4]. Only 2.5–5% of virus carriers develop ATLL after a long period of asymptomatic infection [5]. Molecular studies have shown that viral proteins impair biological activities such as immortalization and IL-2-independent proliferation of T cells induced by the Tax protein [6]. ATLL leukemogenesis can be influenced by genetic and epigenetic alterations, including DNA methylation, and by the host immune system [7]. Despite T-cell immortalization, the long incubation period (>30 years) before ATLL suggests that other genetic alterations besides viral infection contribute to pathogenesis [8].

Certain findings support the transcription of nonprotein coding parts of the mammalian genome [9], which form a network of transcripts interwoven in intricate ways, including noncoding RNAs (ncRNAs). These molecules play important roles in normal biological processes and human diseases such as diabetes [10], aging heart [11], and cancer [12]. In eukaryotes and prokaryotes, small RNAs (sRNAs) regulate posttranscriptional gene expression [13]. The structural and functional complexity of sRNA allows the subdivision of these molecules into regulatory and structural ncRNAs [14]. Examples of structural noncoding RNAs include transfer RNA (tRNA), ribosomal RNA, small nuclear RNAs (snoRNAs), small cytoplasmic RNAs (scRNAs), ribonuclease P (RNase P), mitochondrial RNA processing RNA, signal recognition particle RNA, and telomerase RNA [15]. MicroRNAs (miRNAs/miRs), P-element-induced wimpy testis-interacting RNAs (piRNAs), and long ncRNAs are examples of regulatory ncRNAs [16]. MiRNAs are the most thoroughly studied small ncRNAs. miRNAs are single-stranded RNA molecules with 18 to 25 nucleotides. They have been shown to be important posttranscriptional regulators of gene expression and are important for many cellular processes such as cell growth, differentiation, and apoptosis [17]. HTLV-1 can alter ATLL through dysregulation of host cell miRNAs [18,19]. Some studies suggest that cellular miRNAs play a role in the proliferation and survival of HTLV-I-infected T cells. To date, HTLV-1-infected cell lines have been studied using microarray- and PCR-based approaches to identify and characterize cellular miRNAs [20]. In a recent article published by our group, we reported several sRNA signatures for ATLL and suggested that these signatures could be used as biomarkers for detecting ATLL at an early stage [21]. In a later study, we also found that miR-451-3p was the most downregulated miRNA, suggesting that this miR may be a promising therapeutic target in patients with active ATLL via the AMPK/Notch pathway.

## 2. Case Description

### 2.1. Clinical and Laboratory Materials

In August 2013, a 30-year-old Brazilian woman was admitted to a public hospital in Bahia, northeastern Brazil, because she had been suffering from fever, arthralgia, vomiting, and abdominal pain for two weeks. On physical examination, the patient had cervical, submental, and inguinal lymphadenopathy. She stated that she had no history of swelling of this type and was not taking intravenous drugs. There was evidence of leukemia in her family history. During hospitalization at this time, laboratory findings revealed normal complete blood cells, except for a leukocyte count of 51 × 10^3^/mm^3^, with 2.5 × 10^3^/mm^3^ banded neutrophils, 6.8 × 10^3^/mm^3^ segmented neutrophils, 4.2 × 10^3^/mm^3^ monocytes, and 33 × 10^3^/mm^3^ lymphocytes (Table 1). Laboratory tests also revealed marked hypercalcemia (ionized calcium 8.02 mg/dL). The patient was initially diagnosed with chronic lymphoproliferative disease and was referred to our hospital for further evaluation in September 2013. On admission to our institution, the patient had a leukocyte count of 67.41 × 10^3^/mm^3^ with 60.67 × 10^3^/mm^3^ lymphocytes, Hb 10.1 g/dL, and Hct 32.1%. Morphological and immunophenotypic characteristics of neoplastic cells in peripheral blood showed that 75% of lymphocytes had folded nuclei and condensed chromatin. Flow cytometric analysis of surface markers of lymphocytes in peripheral blood revealed that 66% of pathological T cells expressed CD45 antigen, which expressed the T lymphoid antigens CD3 in low intensity, CD2+, CD5+, and CD4+ with coexpression of CD25+ and loss of CD7 expression. This population was negative for CD8 T-lymphoid antigen, CD13, CD14, CD33, CD64 myelomonocytic antigen, CD19, CD20, CD22 B-lymphoid antigen, and TdT precursor cell CD34. Other peripheral blood findings were as follows: aspartate aminotransferase, 62 U/L; alanine aminotransferase, 40 U/L; alkaline phosphatase, 555 U/L; iron saturation, 72%; lactate dehydrogenase, 2510 U/L; and calcium, 7.2 mg/dL. Symptoms were described as consistent with peripheral T-cell lymphoma unless otherwise noted. Contrast-enhanced computed tomography revealed bilateral pleural effusion and ascites with lymphadenopathy. Serologic analysis revealed a positive reaction against HTLV-1 antibodies, and molecular analysis showed a high proviral load for HTLV-1 tax DNA (4.5 × 10^3^ copies per 1000 cells). A clonal HTLV-1 expansion assay revealed strong evidence of monoclonal T-cell expansion by DNA-based polymerase chain reaction (PCR) of the rearranged γ-T-cell receptor gene (γTCR). Therefore, the patient was diagnosed with acute ATLL with lymphadenopathy according to the Shimoyama classification criteria [22]. She was immediately treated with eight cycles of cyclophosphamide, adriamycin, vincristine, and prednisone (CHOP; 20 September 2013–10 March 2014), followed by maintenance therapy with interferon-α (IFN-α) and zidovudine until June 2019. All drugs were administered intravenously except prednisone, which was taken orally. Five administration schedules for G-CSF were used, starting 24 h after the end of the seventh cycle. Another three administrations of G-CSF, starting 24 h after the end of the eighth cycle, were given because the patient had neutropenia. The selected induction chemotherapy was effective and well tolerated. Bone marrow transplantation was the most challenging in our patient because no matching donor was available. The patient was last seen in September 2022, and she was still in remission with a good prognosis and performance status.

### 2.2. Molecular Analysis

Several studies have shown that sRNA, particularly microRNAs (miRNAs), can have a significant impact on the cellular response to chemotherapeutic agents and that profiling of these entities in peripheral blood could serve as potential biomarkers of response to therapy in certain cancers [23]. Therefore, we decided to generate triplicate sRNA profiles from peripheral blood mononuclear cells (PBMCs) at the time of diagnosis (referred to as J1_1, J1_2, and J1_3) and shortly after maintenance therapy (referred to as J2_1, J2_2, and J2_3) to determine if they were associated with response to therapy. 

**Figure 1:**
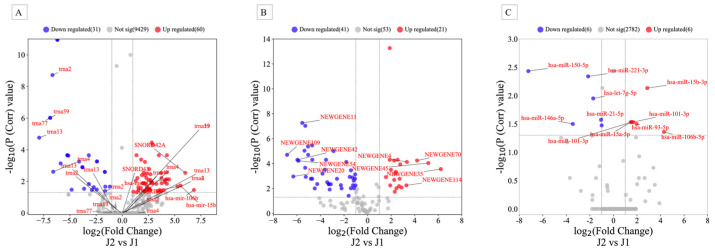
Volcano plots for expressed (**A**) known sRNA, (**B**) novel sRNA, and (**C**) mature miRNAs in peripheral blood mononuclear cells (PBMCs) in J1 (before therapy) and J2 (after maintenance therapy) samples. Red (upregulated) and blue (downregulated) circles indicate genes with significant differences, whereas gray circles represent genes without significant differences.

**Figure 2:**
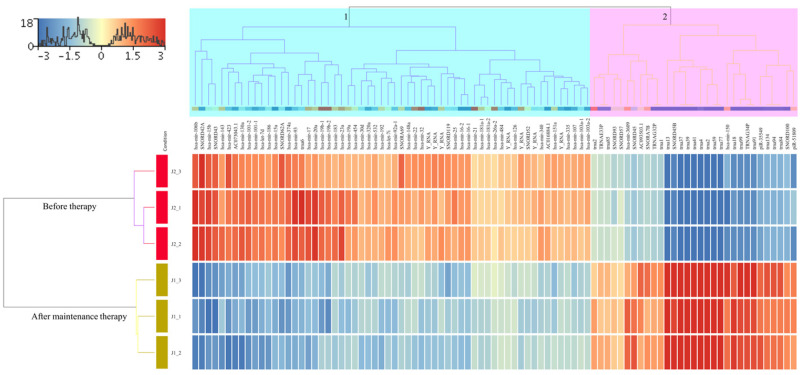
Heatmap showing the results of a hierarchical cluster analysis for the two sample groups and the 91 significantly dysregulated known sRNAs. The sample cluster tree is shown on the left, with the sRNA cluster tree above, forming 2 clusters selected by light green and pink colors. The color scale at the top indicates the relative expression level of sRNA in all samples. Red means that the expression levels are higher than the mean, while blue means that the expression levels are lower than the mean. Each column represents a known sRNA, and each row represents a sample.

Extraction of sRNA, library preparation using the TruSeq sRNA Sample Preparation Kit (Illumina, San Diego, CA, USA), and sequencing on the MiSeq platform were performed according to the manufacturer’s instructions (Illumina, San Diego, CA, USA) and a previous protocol [24,25]. Only high-quality sequencing reads with a Sanger score of 30 or higher were considered for further analysis. Reads were aligned to the whole genome build hg19 using Strand NGS version 3.1 software (Strand Life Science, Bengaluru, India) according to the sRNA alignment and analysis pipeline using default parameters. The distributions of sRNA data in each sample were performed according to the quantile normalization algorithm [26]. In addition, only sRNA sequences that met the minimum read coverage criterion of >5 were considered novel or known sRNA and included in further analyses. The initial analysis showed that the numbers of reads assigned to different gene regions for J1 and J2 were 3,416,083 and 14,018,875, respectively. The median number of reads that passed quality control for J1 and J2 samples were 2,426,809 and 2,413,440, respectively. Before expression analysis, the Shapiro–Wilk method was used to test the normal distribution of the cleaned data (Shapiro–Wilk P > 1.0) in each sample to ensure that only normally distributed data were considered for further analysis. Reads were then normalized using the quantile normalization algorithm [26] with the baseline transformation set to the median value for both samples. We obtained 15,062 of all entities after quantification. Among them were 9945 known genes, 115 novel genes, and 5002 active miRNAs. To identify differentially expressed sRNAs, the read count file of the J1 and J2 files was analyzed using DESeq2, which is included in the Strand NGS version 3.1 package. This analysis identified 91 known sRNAs that were significantly dysregulated before therapy, of which 60 were upregulated and 31 were downregulated, with fold-change values (FC) ≥ 2.0 (Figure 1A, and Supplemental Table S1). The differential expression pattern of these genes in the hierarchical clustering shown in the heatmap in Figure 2 revealed two large clusters in which the J1 and J2 samples could be accurately distinguished.

Of the 115 predicted new sRNAs, 62 were significantly dysregulated between J2 and J1, of which 21 were upregulated and 41 were downregulated with FC values of at least ≥ 2 (Figure 1B, Supplemental Table S2). Hierarchical cluster analysis of these novel sRNAs also showed clear separation of J1 and J2 samples similar to the known sRNAs (Supplemental Figure S1). 

**Figure 3:**
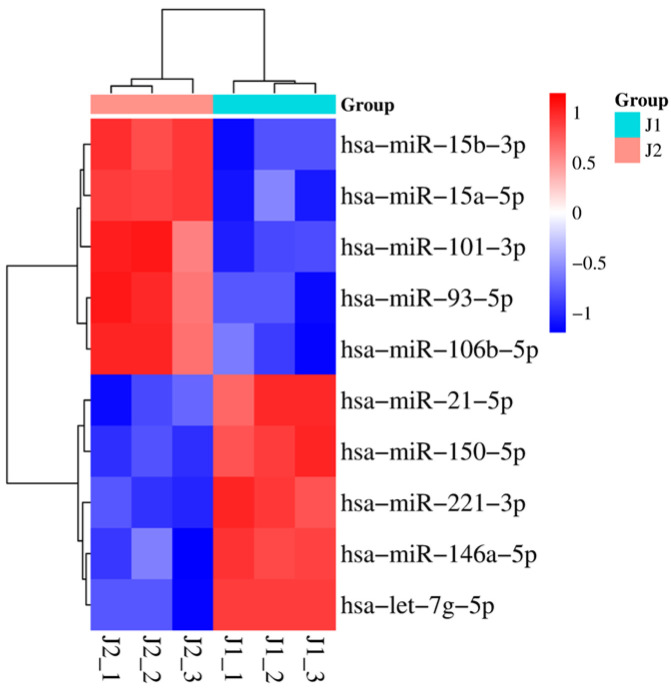
Heatmap showing the results of hierarchical cluster analysis for the two sample groups and the 10 significantly dysregulated mature miRNAs. The sample cluster tree is shown at the top, with the miRNA cluster tree on the left forming two main clusters. The color scale indicates the relative expression level of miRNAs in the J1 and J2 samples.

Five years after therapy, eleven mature miRNAs were differentially dysregulated. Six of these were upregulated, while the other five were downregulated with a fold change ≥ 2. (Figure 1C and Supplemental Table S3). Among the increased miRNAs, miR-106b-5p was 19-fold more altered in the J2 sample than in the J1 sample. On the other hand, miR-150-5p was downregulated more than 150-fold in the J2 sample compared with J1. The nonredundant miRNAs (n = 10) were used to create the heatmap in Figure 3 for unsupervised hierarchical clustering, which clearly distinguishes the two samples.

**Figure 4:**
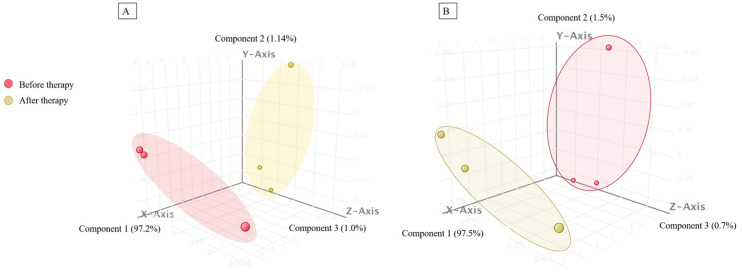
Three-dimensional representation of principal component analysis showing the differences between the J1 (before therapy) and J2 (after maintenance therapy) samples based on (**A**) the 91 significantly deregulated known sRNAs and (**B**) the 11 significantly deregulated miRNAs.

Principal component analysis (PCA) of significantly dysregulated known sRNAs (Figure 4A) and mature miRNAs (Figure 4B) was consistent with the results of hierarchical cluster analysis, which showed a clear separation between J1 and J2.

**Figure 5:**
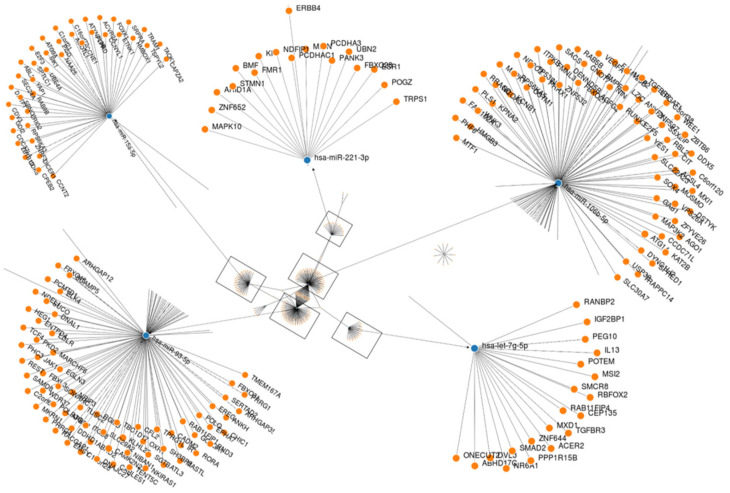
An interaction network between the most deregulated mature miRNAs and their target genes was generated using MirWalk v3. Blue circles represent miRNAs; orange circles represent mRNAs. The more connections there are between miRNAs and genes, the more connections there are in the network.

Gene set enrichment analysis implemented in mirWALK v.3 was used to analyze dysregulated active miRNAs in response to therapy and identify biological networks and functions enriched in the dataset. The analysis predicted 265 target genes for the 10 nonredundant differentially expressed miRNAs (Supplemental Table S4). The miRNA-target gene interaction is shown in Figure 5. All target genes in each dataset were then used to calculate the Reactome, KEGG pathway, and Gene Ontology annotations (GO). The reactome pathways yielded 96 significantly enriched pathways (Corr *p* value ≤ 0.05). Almost all of these pathways were relevant to the phenotype studied and had the highest associated *p* values (Corr). For example, “R-HSA-162582_Signal transduction” was the most strongly predicted pathway enriched with 88 target genes (Supplemental Table S5). KEGG pathway analysis revealed significant enrichment of 56 pathways (FDR < 0.05), most of which were involved in cancer. Endocrine resistance, human T-cell leukemia virus 1 infection, pathways in cancer, and microRNAs in cancer, were the most significant KEGG pathways.

**Figure 6:**
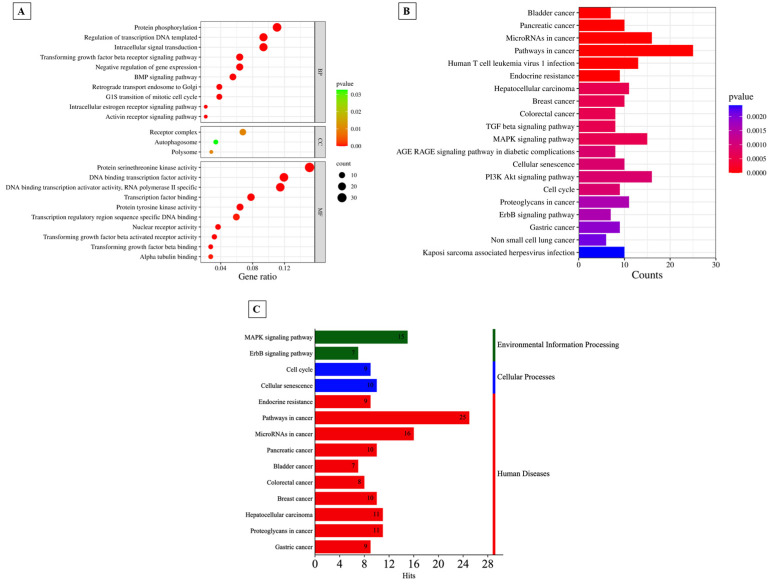
(**A**)Top 10 items of GO BP, GO MF, and all significantly computed GO CC shown in the bubble plot according to adjusted *p* value. (**B**) Classification of the pathways into 3 categories: environmental information processing, cellular processes, and human diseases. (C) Histogram of KEGG pathway annotation of the target genes in ATLL after maintenance therap.

As shown in Supplemental Table S5, annotation of GO revealed that 74 terms from GO were significantly (FDR < 0.05) associated with biological processes (BP), three with cellular components (CC), and 30 with molecular functions (MF). The top ten BP, MF, and all 3 CC terms are shown in Figure 6A. Similarly, the top 20 KEGG pathways are shown in Figure 6B. The results suggested that the differentially expressed genes were mainly involved in human-disease-related pathways, mainly cancer (Figure 6C).

## 3. Discussion

To our knowledge, this is the first case study investigating miRNAs in ATLL patients who received CHOP chemotherapy followed by IFN-α and zidovudine as maintenance therapy. Patients with aggressive forms of ATLL (acute and lymphoma) have the poorest prognosis, with a median survival of only 6–10 months, even with intensive chemotherapy [27]. The worst prognosis results from inherent chemoresistance, significant tumor burden, hypercalcemia, and/or recurrent infectious problems due to significant immunodeficiency [28]. Another possible explanation is that miRNAs control the expression of proteins that cause treatment failure. This allows the cancer cells to acquire the desired properties [29]. In the present study, we examined the expression of sRNA in PBMCs from HTLV-1-infected patients with acute ATLL treated with IFN-α and CHOP chemotherapy. Comparison of sRNA levels after the maintenance phase of therapy and before chemotherapy revealed 91 significantly dysregulated known sRNAs, 62 predicted new sRNAs, and 11 mature miRNAs. Most of the target genes predicted in this study were involved in various BPs, of which the G1S transition of the mitotic cell cycle, DNA-templated transcription, and the transforming growth factor beta receptor signaling pathway were the most important. Five proteins, namely PPP6C, CDK6, CCNE1, E2F3, and CCND1, were targeted by hsa-miR-15b-3p, miR-93-5p, and miR-106b-5p and are collectively known to interact and regulate the G1 to S transition in mitotic cells by promoting the release of E2F transcription factors and activating gene expression necessary for DNA replication and cell cycle progression [30,31]. Available data from the literature suggest that overexpression of p21^WAF1/CIP1^ is associated with G1 arrest and that this protein is regulated by both p53-dependent and p53-independent pathways [32]. Thus, activation of p53 is an important suppressor that ensures that cells are arrested in G1 after DNA damage so that repair and replication can proceed normally. In ATLL, there are both Tax-dependent and Tax-independent mechanisms for inactivation of p53 functions [33], and genetic inactivation of p53 was found in 30% of acute cases [34]. Previous studies have shown that Tax inactivates p53 via either activation of the CREB [35] or NF-κB pathway [36]. Datta and colleagues [33] demonstrated that the inactivation of p53 can be reversed through AZT treatment in T-cell leukemia-virus-I-infected cells. This effect was observed both in laboratory experiments (in vitro) and in patients with ATLL (in vivo). The treatment resulted in the inhibition of telomerase activity, gradual shortening of telomeres, and an increase in p14ARF expression. As a result, the transcription of tumor suppressor p53-dependent was stabilized and reactivated, leading to enhanced expression of the cyclin-dependent kinase inhibitor p21^Waf1^ and accumulation of p27^kip1^. These changes ultimately induced cellular senescence and caused the death of tumor cells. Based on these results, it is plausible to hypothesize that IFNα/AZT therapy activates the p53 pathway in our patient. This activation of the p53 pathway upregulates the expression of miR-106b, which works together with the antitumor effect of p53, potentially enhancing its effectiveness against leukemic cells.

In our current case study, among the five nonredundant miRNAs upregulated after maintenance therapy, miR-106b-5p was shown to target tumor protein 53-induced nuclear protein 1 (TP53INP1), whose activity determines cellular survival and proliferation. Of note, miR-106b-5p was the most upregulated miRNA and was identified, together with miR-93-5p, as the one targeting many target genes. In addition, miR-93-5p, together with miR-130b, was reported to interact in a regulatory manner with TP53INP1 in HTLV-I-transformed cells [19]. Another important BP in ATLL was the transforming growth factor beta receptor (TGF-β) signaling pathway. In this study, hsa-miR-93-5p, miR-150-5p, miR-106b-5p, and hsa-let-7g-5p were predicted to target TP53, SMAD2, TGFBR2, TGFBR3, and ITGB8. These proteins collectively contribute to the TGF-β signaling pathway, which is highly intricate and tightly regulated. Their interactions and functions within the pathway are essential for proper cellular responses to TGF-β signaling [37,38,39]. Several studies have shown that abnormal expression of miRNAs can affect TGF-β signaling [40], which plays a critical role in ATLL development and progression [41,42]. For example, miR-26b was shown to downregulate the expression of SMAD-4, a negative regulator of TGF-β signaling, thereby promoting TGF-β signaling in affected cells [43] including leukemic cells [44]. One of the target genes involved in the regulation of TGF-β signaling is TGF-β R2 [45], which was also targeted by miR-106b-5p in our study. Lee et al. [46] recently demonstrated that the expression of miR-106b-5p can predict the progression and recurrence of breast cancer in situ via the TGF-β pathway. Elevated levels of TGF-β-producing T cells and regulatory T cells play a critical role as a risk factor in the onset of ATLL [41]. Yamagishi et al. [47] conducted a study revealing a substantial increase in TGF-β-producing T cells in both freshly isolated and long-term cultured T cells derived from ATLL patients. The overexpression of TGF-β during ATLL pathogenesis is believed to be primarily attributed to HBZ [48]. Based on the aforementioned findings, although not specifically examined in this study, it can be speculated that the substantial upregulation of miR-106b-5p following maintenance therapy may contribute to the inactivation of TGF-β signaling. This effect could be achieved by miR-106b-5p directly targeting TβR II, which is responsible for initiating multiple TGF-β signaling pathways [49]. If this hypothesis is confirmed in future investigations, the dysregulation of miR-106b-5p may add an additional level of control to suppress the functions of TGF-β signaling pathways, thereby mitigating the malignant proliferation observed in ATLL.

Previous studies have shown that arsenic trioxide in combination with IFN-α induces cell cycle arrest and apoptosis in HTLV-I-infected and freshly isolated leukemia cells from ATLL patients [50] by rapidly silencing NF-κB signaling and delaying cell cycle-associated gene silencing due to Tax degradation by the proteasome [51,52]. Our results support these studies and suggest that the combination of chemotherapeutic agents and IFN-α likely affects miRNA expression and promotes the expression of target genes that induce cell cycle arrest and apoptosis.

After the maintenance phase of therapy, the most elevated miRNAs were miR-106-5p and miR-15b-3p, and the most downregulated were miR-150-5p and miR-146a-5p. Overexpression of miR-106b has been observed in numerous tumor types and regulates cell proliferation, migration, invasion, and metastasis. Breast cancer [53], prostate cancer [54], lung cancer [55], gastric cancer [56], colorectal carcinoma [57], hepatocellular carcinoma [58], and squamous cell carcinoma of the esophagus [59] have all been associated with abnormal miR-106b expression. In the context of leukemia, in a recent study, Moussa Agha et al. [60] investigated the circulating miRNA profile in 27 patients with bone marrow acute myeloid leukemia (AML) at the time of diagnosis and at the first complete remission after treatment compared with 11 healthy donors. The results showed that several miRNAs, including miR-106b, were deregulated among the three groups and were associated with tumor progression and immunosuppression. The miRNA expression profile data of B-cell acute lymphoblastic leukemia (ALL) showed that miR-106b-5p was the most downregulated miRNA (fold-change, 1509.5) [61]. Sampath et al. [62] revealed the involvement of miRNA-106b in translational suppression of ITCH and demonstrated reactivation of p73-dependent apoptosis in primary chronic lymphocytic leukemia (CLL) cells after expression of miR-106b. MiR-106b expression has also been investigated in prognostic studies of leukemia. High miR-106b expression has been associated with lower overall survival, disease-free survival, and relapse [63]. For example, patients with AML who express more miR-106b have poorer overall survival and are more resistant to therapy. Another study showed that the expression of the miR-106b-25 cluster is increased in recurrent AML in pediatric patients caused by mixed gene rearrangement, suggesting that miR-106b-25 is closely associated with the recurrence of AML [64]. Available data from various studies suggest that miR-106b may be a potential biomarker for tumor detection, prognosis assessment, and therapeutic targets in various cancers [65,66,67,68]. However, such studies are rare in leukemia, except for a single study by Zhang et al. [63] who investigated the biological role and underlying mechanisms of miR-106b-25 in drug resistance in leukemia. Their results showed that miR-106-25 was associated with drug resistance in AML and disease prognosis. Further experimental investigation in the same study revealed that the biological effects of the miR-106b-25 cluster on leukemic cell proliferation, chemoresistance, and apoptosis are mediated through the regulation of the apoptotic pathway. Consistent with the existing evidence linking miR-106b-5p to AML, ALL, CLL, and other cancers, our hypothesis suggests that miR-106b-5p plays a role in the therapeutic mechanism of ATLL. Furthermore, we propose that elevated expression of this miRNA is correlated with a favorable prognosis.

miR-15b was the second most expressed miR in our patient after completion of maintenance therapy. It has been reported that the expression level of miR-15b in PBMCs correlates with baseline blood glucose levels and may serve as a useful indicator of diabetes [69]. miR-15b-3p has also been extensively studied in the context of cancer and has shown both oncogenic and tumor suppressive functions depending on the cancer type and cellular context [70,71,72]. Lu et al. [73] showed that miR-15b inhibits the proliferation, migration, and invasion of thyroid cancer cells by regulating Bcl-2. In contrast, miR-15b promotes cancer growth in other malignancies. Thus, miR-15b overexpression increased sunitinib resistance and promoted cell survival and invasion in renal cell cancer [73]. Therefore, it was hypothesized that the function of miR-15b may vary depending on the type of cancer. Recently, Sun et al. [74] demonstrated that overexpression of miR-15b promotes apoptosis and disrupts the cell cycle of gliomas by targeting cyclin D1 (CCND1). They concluded that CCND1 represents a novel therapeutic option for the treatment of gliomas. CCND1 is associated with tumor malignancy and poor prognosis and has been shown to be involved in miRNA regulatory networks and their downstream target genes [75]. Close inspection of the interaction between miRNAs and target genes in our patient revealed that CCND1 was targeted by miR-106b-5p, miR-93-5p, and miR-15b. Thus, it is possible that the direct influence of these miRs on CCND1 inhibits the proliferation of leukemic cells by stopping their cycle progression and inducing apoptosis, suggesting that these miRs, including miR-15b, may have a suppressive function in ATLL. 

miR-150 has been found to be dysregulated in various types of solid cancers and hematologic malignancies [76,77]. Several studies have shown that miR-150 affects oncogenes and/or tumor suppressor genes and that this has both a curative and malignant effect on tumors [78,79]. In 2007, Fulci et al. [80] found that miR-150 was significantly increased in CD19+ B cells from patients with CLL compared with healthy donors. In contrast to CLL, miR-150 was downregulated in CD34+ or mononuclear cells from patients with chronic myeloid leukemia (CML) compared with healthy donors [81]. Several studies have found that miR-150 expression is significantly lower in pediatric acute lymphoblastic leukemia (ALL) cells than the control cells [82,83]. These results suggest that downregulation of miR-150 is associated with a higher probability of disease recurrence in ALL patients, suggesting a potential favorable prognostic effect of this miR in ALL. One of the most predicted targets of miR-150 was the transcription factor MYB, which is critical for lymphocyte development [84]. MYB plays a central role in regulating hematopoietic cell development and turnover and is deregulated in cancer cells [85]. The HTLV-1 proteins Tax and HBZ were shown to be responsible for alterations in MYB function and expression in HTLV-1-infected cell lines [86]. Nakano et al. [87] reported that the expression of MYB is significantly higher in ATLL samples than in CD4+ T-cell controls. Moreover, in the same study, MYB and its splicing variant MYB-9A were found to be able to activate the NF-κB signaling pathway, and knockdown of MYB or MYB-9A induced apoptosis of ATLL cells. In another study, the oncogenic transcription factor c-Myc (Myc) was shown to inhibit the expression of numerous miRNAs, including miR-150 [88]. Further investigation revealed a regulatory loop in which Myc promotes the development of LIN28, an RNA-binding protein that inhibits the maturation of multiple miRNAs, including miR-150 [78,88,89]. This consequently results in dysregulation of oncogenes such as MYB and FLT3, which are normally targeted by miR-150 [90]. The above data clearly suggest that the downregulation of miR-150 is associated with poor prognosis in ATL and other leukemic diseases. However, this is not true for our patient, who had a good prognosis after completion of therapy, although miR-150-5p was significantly downregulated and its target gene MYB was predicted. One conceivable interpretation of the low miR-150-5p levels could be that chemotherapy simply reduces the leukemic cells and extracellular vehicles (EVs) into which miR-150-5p is actively exported [91], leading to an apparent reduction in its burden.

The dysregulation of miR-146a-5p has been implicated in the pathogenesis and progression of different types of cancer tissues. Overexpression of miR-146a has been found in cervical cancer cells [92]) and in Burkitt’s lymphoma [93], whereas low expression has been described in thyroid carcinomas [94] and prostate cancer [95]. It is known that miR-146a plays an essential role in several viral infections, whereas its role in other infections, especially oncoviruses, is complex [96]. For example, Tomita et al. [97] demonstrated that miR-146a is highly expressed in HTLV-1-infected T-cell lines and is directly induced by Tax via activation of NF-κB signaling. Pichler et al. [18] also reported upregulation of miR-146a along with other 3 miRs in HTLV-1-transformed cells. In our patient, miR-146a was markedly downregulated after maintenance therapy, likely due to the induction of apoptosis by IFNα/AZT treatment in the patient’s ATLL cells [98]. This downregulation leads to suppression of NF-κB activation. However, the mechanism underlying the prognostic therapeutic role of miR-146a in acute ATLL remains to be fully elucidated.

There were several limitations in our study. First, it was a single case study, which limits the generalizability of the findings. Additionally, we did not conduct any additional molecular biology experiments to validate the differentially expressed miRNAs, their potential mechanisms in regulating target genes, and their functions. Last, these observations need to be validated in larger ATLL cohorts to establish their prognostic significance.

Altered miRNA expression patterns have the potential to function as biomarkers for leukemia diagnosis, prognosis prediction, and therapy response evaluation, as evidenced in our patient. Therefore, therapies based on miRNAs hold promising prospects for leukemia treatment. By overcoming the challenges related to specificity, delivery, and functional complexity and by utilizing personalized medicine and combination therapies, we can establish the groundwork for future treatment strategies in patients with leukemia.

In conclusion, we identified 11 miRNAs that may play a role in the therapeutic mechanism of acute ATLL. These miRNAs might exert their effects by regulating various functions, especially the G1S transition of the mitotic cell cycle. Moreover, these 11 miRNAs might be involved in various signaling pathways. On the one hand, PPP6C, CDK6, CCNE1, E2F3, and CCND1 might serve as crucial regulators in the G1S transition, while TP53, SMAD2, TGFBR2, TGFBR3, and ITGB8, on the other hand, might play a role in the TGF-β signaling pathway. However, further studies are needed to fully understand the specific functions of these miRNAs in HTLV-1-associated acute ATLL therapy and prognosis. Moreover, experimental validation is needed to identify the target genes of these miRNAs.

## Figures and Tables

**Table 1:** Timeline of treatment course and clinical information.

Laboratory Tests	Diagnosis		Induction Chemotherapy	Maintenance Therapy			Follow Up		
			September 2013–March 2014						
	Aug/2013	Sep/2013	2014	2015	2016	2017	2018	2019	2022
HTLV-1/2		Reactive				Reactive			
T cell clonality test		Monoclonal population							
Platelets (Thousand/mm^3^)	360	218							370
MPV (fL)	9.1	12.3							10.7
Red Blood Cell Count (million/mm^3^)	5.1	3.64	4.45	4.23	4.49	4.78	4.89	4.92	4.49
Hemoglobin (g/dL)	14.6	10.1	11.7	11.50	12.3	13.3	13.3	13.7	12.8
Hematocrit (%)	45.6	32.1	35.5	35.3	37.3	39.7	40.7	40.9	38.1
MCV (fL)	89.4	88.2	79.8	83.5	83.1	83.1	83.2	83.1	84.9
MCH (pg)	28.6	27.7	26.3	27.2	27.4	27.8	27.2	27.8	28.5
MCHC (g/dL)	32	31.5	33	32.6	33	33.5	32.7	33.5	33.6
RDW-VC (%)		15.8	13.5	14.5	13.7	13.9	13.3	13.2	13.3
RDW-SD (fL)		49.1	39.1	44.4	41.6	41.7	40.1	39.9	40.3
Leukocytes (Thousand/mm^3^)	51	67.41	3.52	4.09	11.94	5.3	4.6	6.97	8.45
Banded neutrophils (Thousand/mm^3^)	2.5								
Segmented neutrophils (Thousand/mm^3^)	6.8	6.07	1.35	1.8	7.6	2.18	1.64	4.06	5.6
Eosinophils (Thousand/mm^3^)	0.2	0	0.04	0.07	0	0.05	0.06	0.05	0.1
Basophils (Thousand/mm^3^)	4	0	0.02	0.01	0.01	0.04	0.02	0.04	0
Lymphocytes (Thousand/mm^3^)	33	60.67	1.8	1.76	3.4	2.33	2.47	2.39	2.2
Monocytes (Thousand/mm^3^)	4.2	0.67	0.31	0.45	0.9	0.7	0.41	0.43	0.6

HTLV-1/2: Human T-lymphotropic viruses, type I/II, MPV: mean platelet volume, MCV: mean corpuscular volume, MCH: mean corpuscular hemoglobin. MCHC: mean corpuscular hemoglobin concentration, RDW: red cell distribution width.

## Data Availability

All sequence data described here are available in the online Zenodo repository https://doi.org/10.5281/zenodo.8080086.

## Supplementary Materials

The supporting information can be downloaded at: https://www.mdpi.com/article/10.3390/ijms241310643/s1.

## References

[1] Poiesz B.J., Ruscetti F.W., Gazdar A.F., Bunn P.A., Minna J.D., Gallo R.C. (1980). Detection and isolation of type C retrovirus particles from fresh and cultured lymphocytes of a patient with cutaneous T-cell lymphoma. Proc. Natl. Acad. Sci. USA.

[2] Gessain A., Cassar O. (2012). Epidemiological Aspects and World Distribution of HTLV-1 Infection. Front. Microbiol..

[3] Verdonck K., Gonzalez E., Van Dooren S., Vandamme A.M., Vanham G., Gotuzzo E. (2007). Human T-lymphotropic virus 1: Recent knowledge about an ancient infection. Lancet. Infect. Dis..

[4] Franchini G., Ambinder R.F., Barry M. (2000). Viral Disease in Hematology. Hematol. Am. Soc. Hematol. Educ. Program.

[5] Murphy E.L., Hanchard B., Figueroa J.P., Gibbs W.N., Lofters W.S., Campbell M., Goedert J.J., Blattner W.A. (1989). Modelling the risk of adult T-cell leukemia/lymphoma in persons infected with human T-lymphotropic virus type I. Int. J. Cancer.

[6] Higuchi M., Fujii M. (2009). Distinct functions of HTLV-1 Tax1 from HTLV-2 Tax2 contribute key roles to viral pathogenesis. Retrovirology.

[7] Yoshida M., Seiki M., Yamaguchi K., Takatsuki K. (1984). Monoclonal integration of human T-cell leukemia provirus in all primary tumors of adult T-cell leukemia suggests causative role of human T-cell leukemia virus in the disease. Proc. Natl. Acad. Sci. USA.

[8] Nagata Y., Kontani K., Enami T., Kataoka K., Ishii R., Totoki Y., Kataoka T.R., Hirata M., Aoki K., Nakano K. (2016). Variegated RHOA mutations in adult T-cell leukemia/lymphoma. Blood.

[9] Djebali S., Davis C.A., Merkel A., Dobin A., Lassmann T., Mortazavi A., Tanzer A., Lagarde J., Lin W., Schlesinger F. (2012). Landscape of transcription in human cells. Nature.

[10] Higuchi C., Nakatsuka A., Eguchi J., Teshigawara S., Kanzaki M., Katayama A., Yamaguchi S., Takahashi N., Murakami K., Ogawa D. (2015). Identification of circulating miR-101, miR-375 and miR-802 as biomarkers for type 2 diabetes. Metab. Clin. Exp..

[11] van Rooij E., Sutherland L.B., Qi X., Richardson J.A., Hill J., Olson E.N. (2007). Control of stress-dependent cardiac growth and gene expression by a microRNA. Science.

[12] Zhang W.C., Chin T.M., Yang H., Nga M.E., Lunny D.P., Lim E.K., Sun L.L., Pang Y.H., Leow Y.N., Malusay S.R. (2016). Tumour-initiating cell-specific miR-1246 and miR-1290 expression converge to promote non-small cell lung cancer progression. Nat. Commun..

[13] Farazi T.A., Juranek S.A., Tuschl T. (2008). The growing catalog of small RNAs and their association with distinct Argonaute/Piwi family members. Development.

[14] Mattick J.S., Makunin I.V. (2006). Non-coding RNA. Hum. Mol. Genet..

[15] Ambros V., Bartel B., Bartel D.P., Burge C.B., Carrington J.C., Chen X., Dreyfuss G., Eddy S.R., Griffiths-Jones S., Marshall M. (2003). A uniform system for microRNA annotation. Rna.

[16] Li J., Wu B., Xu J., Liu C. (2014). Genome-wide identification and characterization of long intergenic non-coding RNAs in Ganoderma lucidum. PLoS ONE.

[17] Xu P., Vernooy S.Y., Guo M., Hay B.A. (2003). The Drosophila microRNA Mir-14 suppresses cell death and is required for normal fat metabolism. Curr. Biol. CB.

[18] Pichler K., Schneider G., Grassmann R. (2008). MicroRNA miR-146a and further oncogenesis-related cellular microRNAs are dysregulated in HTLV-1-transformed T lymphocytes. Retrovirology.

[19] Yeung M.L., Yasunaga J., Bennasser Y., Dusetti N., Harris D., Ahmad N., Matsuoka M., Jeang K.T. (2008). Roles for microRNAs, miR-93 and miR-130b, and tumor protein 53-induced nuclear protein 1 tumor suppressor in cell growth dysregulation by human T-cell lymphotrophic virus 1. Cancer Res..

[20] Huntzinger E., Izaurralde E. (2011). Gene silencing by microRNAs: Contributions of translational repression and mRNA decay. Nat. Rev. Genet..

[21] Nascimento A., Valadao de Souza D.R., Pessoa R., Pietrobon A.J., Nukui Y., Pereira J., Casseb J., Penalva de Oliveira A.C., Loureiro P., da Silva Duarte A.J. (2021). Global expression of noncoding RNome reveals dysregulation of small RNAs in patients with HTLV-1-associated adult T-cell leukemia: A pilot study. Infect. Agents Cancer.

[22] Shimoyama M. (1991). Diagnostic criteria and classification of clinical subtypes of adult T-cell leukaemia-lymphoma. A report from the Lymphoma Study Group (1984–87). Br. J. Haematol..

[23] Si W., Shen J., Zheng H., Fan W. (2019). The role and mechanisms of action of microRNAs in cancer drug resistance. Clin. Epigenetics.

[24] Clissa P.B., Pessôa R., Ferraz K.F., de Souza D.R., Sanabani S.S. (2016). Data on global expression of non-coding RNome in mice gastrocnemius muscle exposed to jararhagin, snake venom metalloproteinase. Data Brief.

[25] Valadao de Souza D.R., Pessoa R., Nascimento A., Nukui Y., Pereira J., Casseb J., Penalva de Oliveira A.C., da Silva Duarte A.J., Clissa P.B., Sanabani S.S. (2020). Small RNA profiles of HTLV-1 asymptomatic carriers with monoclonal and polyclonal rearrangement of the T-cell antigen receptor gamma-chain using massively parallel sequencing: A pilot study. Oncol. Lett..

[26] Hansen K.D., Irizarry R.A., Wu Z. (2012). Removing technical variability in RNA-seq data using conditional quantile normalization. Biostatistics.

[27] Yamada Y., Tomonaga M., Fukuda H., Hanada S., Utsunomiya A., Tara M., Sano M., Ikeda S., Takatsuki K., Kozuru M. (2001). A new G-CSF-supported combination chemotherapy, LSG15, for adult T-cell leukaemia-lymphoma: Japan Clinical Oncology Group Study 9303. Br. J. Haematol..

[28] Bazarbachi A., Suarez F., Fields P., Hermine O. (2011). How I treat adult T-cell leukemia/lymphoma. Blood.

[29] Mesrian Tanha H., Mojtabavi Naeini M., Rahgozar S., Moafi A., Honardoost M.A. (2016). Integrative computational in-depth analysis of dysregulated miRNA-mRNA interactions in drug-resistant pediatric acute lymphoblastic leukemia cells: An attempt to obtain new potential gene-miRNA pathways involved in response to treatment. Tumour Biol. J. Int. Soc. Oncodev. Biol. Med..

[30] Ooi A., Oyama T., Nakamura R., Tajiri R., Ikeda H., Fushida S., Dobashi Y. (2017). Gene amplification of CCNE1, CCND1, and CDK6 in gastric cancers detected by multiplex ligation-dependent probe amplification and fluorescence in situ hybridization. Hum. Pathol..

[31] Inoshita S., Terada Y., Nakashima O., Kuwahara M., Sasaki S., Marumo F. (1999). Regulation of the G1/S transition phase in mesangial cells by E2F1. Kidney Int..

[32] el-Deiry W.S., Harper J.W., O’Connor P.M., Velculescu V.E., Canman C.E., Jackman J., Pietenpol J.A., Burrell M., Hill D.E., Wang Y. (1994). WAF1/CIP1 is induced in p53-mediated G1 arrest and apoptosis. Cancer Res..

[33] Datta A., Bellon M., Sinha-Datta U., Bazarbachi A., Lepelletier Y., Canioni D., Waldmann T.A., Hermine O., Nicot C. (2006). Persistent inhibition of telomerase reprograms adult T-cell leukemia to p53-dependent senescence. Blood.

[34] Sakashita A., Hattori T., Miller C.W., Suzushima H., Asou N., Takatsuki K., Koeffler H.P. (1992). Mutations of the p53 gene in adult T-cell leukemia. Blood.

[35] Ariumi Y., Kaida A., Lin J.Y., Hirota M., Masui O., Yamaoka S., Taya Y., Shimotohno K. (2000). HTLV-1 tax oncoprotein represses the p53-mediated trans-activation function through coactivator CBP sequestration. Oncogene.

[36] Pise-Masison C.A., Mahieux R., Jiang H., Ashcroft M., Radonovich M., Duvall J., Guillerm C., Brady J.N. (2000). Inactivation of p53 by human T-cell lymphotropic virus type 1 Tax requires activation of the NF-kappaB pathway and is dependent on p53 phosphorylation. Mol. Cell. Biol..

[37] Mu D., Cambier S., Fjellbirkeland L., Baron J.L., Munger J.S., Kawakatsu H., Sheppard D., Broaddus V.C., Nishimura S.L. (2002). The integrin alpha(v)beta8 mediates epithelial homeostasis through MT1-MMP-dependent activation of TGF-beta1. J. Cell Biol..

[38] Ungefroren H., Groth S., Sebens S., Lehnert H., Gieseler F., Fandrich F. (2011). Differential roles of Smad2 and Smad3 in the regulation of TGF-beta1-mediated growth inhibition and cell migration in pancreatic ductal adenocarcinoma cells: Control by Rac1. Mol. Cancer.

[39] Batlle E., Massague J. (2019). Transforming Growth Factor-beta Signaling in Immunity and Cancer. Immunity.

[40] Nabhan M., Louka M.L., Khairy E., Tash F., Ali-Labib R., El-Habashy S. (2017). MicroRNA-181a and its target Smad 7 as potential biomarkers for tracking child acute lymphoblastic leukemia. Gene.

[41] Kim S.J., Kehrl J.H., Burton J., Tendler C.L., Jeang K.T., Danielpour D., Thevenin C., Kim K.Y., Sporn M.B., Roberts A.B. (1990). Transactivation of the transforming growth factor beta 1 (TGF-beta 1) gene by human T lymphotropic virus type 1 tax: A potential mechanism for the increased production of TGF-beta 1 in adult T cell leukemia. J. Exp. Med..

[42] Niitsu Y., Urushizaki Y., Koshida Y., Terui K., Mahara K., Kohgo Y., Urushizaki I. (1988). Expression of TGF-beta gene in adult T cell leukemia. Blood.

[43] Dong N., Xu B., Benya S.R., Tang X. (2014). MiRNA-26b inhibits the proliferation, migration, and epithelial-mesenchymal transition of lens epithelial cells. Mol. Cell. Biochem..

[44] Marquez M.E., Sernbo S., Payque E., Uria R., Tosar J.P., Querol J., Berca C., Uriepero A., Prieto D., Alvarez-Saravia D. (2022). TGF-beta/SMAD Pathway Is Modulated by miR-26b-5p: Another Piece in the Puzzle of Chronic Lymphocytic Leukemia Progression. Cancers.

[45] Kim B.G., Malek E., Choi S.H., Ignatz-Hoover J.J., Driscoll J.J. (2021). Novel therapies emerging in oncology to target the TGF-beta pathway. J. Hematol. Oncol..

[46] Lee J., Kim H.E., Song Y.S., Cho E.Y., Lee A. (2019). miR-106b-5p and miR-17-5p could predict recurrence and progression in breast ductal carcinoma in situ based on the transforming growth factor-beta pathway. Breast Cancer Res. Treat..

[47] Yamagishi M., Watanabe T. (2012). Molecular hallmarks of adult T cell leukemia. Front. Microbiol..

[48] Zhao T., Satou Y., Sugata K., Miyazato P., Green P.L., Imamura T., Matsuoka M. (2011). HTLV-1 bZIP factor enhances TGF-beta signaling through p300 coactivator. Blood.

[49] Luo K., Lodish H.F. (1997). Positive and negative regulation of type II TGF-beta receptor signal transduction by autophosphorylation on multiple serine residues. EMBO J..

[50] Bazarbachi A., El-Sabban M.E., Nasr R., Quignon F., Awaraji C., Kersual J., Dianoux L., Zermati Y., Haidar J.H., Hermine O. (1999). Arsenic trioxide and interferon-alpha synergize to induce cell cycle arrest and apoptosis in human T-cell lymphotropic virus type I-transformed cells. Blood.

[51] El-Sabban M.E., Nasr R., Dbaibo G., Hermine O., Abboushi N., Quignon F., Ameisen J.C., Bex F., de The H., Bazarbachi A. (2000). Arsenic-interferon-alpha-triggered apoptosis in HTLV-I transformed cells is associated with tax down-regulation and reversal of NF-kappa B activation. Blood.

[52] Nasr R., Rosenwald A., El-Sabban M.E., Arnulf B., Zalloua P., Lepelletier Y., Bex F., Hermine O., Staudt L., de The H. (2003). Arsenic/interferon specifically reverses 2 distinct gene networks critical for the survival of HTLV-1-infected leukemic cells. Blood.

[53] Li N., Miao Y., Shan Y., Liu B., Li Y., Zhao L., Jia L. (2017). MiR-106b and miR-93 regulate cell progression by suppression of PTEN via PI3K/Akt pathway in breast cancer. Cell Death Dis..

[54] Hudson R.S., Yi M., Esposito D., Glynn S.A., Starks A.M., Yang Y., Schetter A.J., Watkins S.K., Hurwitz A.A., Dorsey T.H. (2013). MicroRNA-106b-25 cluster expression is associated with early disease recurrence and targets caspase-7 and focal adhesion in human prostate cancer. Oncogene.

[55] Yu S., Qin X., Chen T., Zhou L., Xu X., Feng J. (2017). MicroRNA-106b-5p regulates cisplatin chemosensitivity by targeting polycystic kidney disease-2 in non-small-cell lung cancer. Anti-Cancer Drugs.

[56] Yu D., Shin H.S., Lee Y.S., Lee Y.C. (2014). miR-106b modulates cancer stem cell characteristics through TGF-beta/Smad signaling in CD44-positive gastric cancer cells. Lab. Investig. A J. Technol. Methods Pathol..

[57] Peng Q., Shen Y., Zhao P., Cheng M., Zhu Y., Xu B. (2020). Biomarker roles identification of miR-106 family for predicting the risk and poor survival of colorectal cancer. BMC Cancer.

[58] Li Y., Tan W., Neo T.W., Aung M.O., Wasser S., Lim S.G., Tan T.M. (2009). Role of the miR-106b-25 microRNA cluster in hepatocellular carcinoma. Cancer Sci..

[59] Kan T., Sato F., Ito T., Matsumura N., David S., Cheng Y., Agarwal R., Paun B.C., Jin Z., Olaru A.V. (2009). The miR-106b-25 polycistron, activated by genomic amplification, functions as an oncogene by suppressing p21 and Bim. Gastroenterology.

[60] Moussa Agha D., Rouas R., Najar M., Bouhtit F., Naamane N., Fayyad-Kazan H., Bron D., Meuleman N., Lewalle P., Merimi M. (2020). Identification of Acute Myeloid Leukemia Bone Marrow Circulating MicroRNAs. Int. J. Mol. Sci..

[61] Lin X.C., Liu X.G., Zhang Y.M., Li N., Yang Z.G., Fu W.Y., Lan L.B., Zhang H.T., Dai Y. (2017). Integrated analysis of microRNA and transcription factor reveals important regulators and regulatory motifs in adult B-cell acute lymphoblastic leukemia. Int. J. Oncol..

[62] Sampath D., Calin G.A., Puduvalli V.K., Gopisetty G., Taccioli C., Liu C.G., Ewald B., Liu C., Keating M.J., Plunkett W. (2009). Specific activation of microRNA106b enables the p73 apoptotic response in chronic lymphocytic leukemia by targeting the ubiquitin ligase Itch for degradation. Blood.

[63] Zhang M., Xiao F., Li Y., Chen Z., Zhang X., Xing W., Yuan W., Zhou Y. (2021). MiR-106b-25 Promotes Chemoresistance in Acute Myeloid Leukemia Via Abolishing Multiple Apoptotic Pathways. Blood.

[64] Verboon L.J., Obulkasim A., de Rooij J.D., Katsman-Kuipers J.E., Sonneveld E., Baruchel A., Trka J., Reinhardt D., Pieters R., Cloos J. (2016). MicroRNA-106b~25 cluster is upregulated in relapsed MLL-rearranged pediatric acute myeloid leukemia. Oncotarget.

[65] Zheng R., Pan L., Gao J., Ye X., Chen L., Zhang X., Tang W., Zheng W. (2015). Prognostic value of miR-106b expression in breast cancer patients. J. Surg. Res..

[66] Gorur A., Balci Fidanci S., Dogruer Unal N., Ayaz L., Akbayir S., Yildirim Yaroglu H., Dirlik M., Serin M.S., Tamer L. (2013). Determination of plasma microRNA for early detection of gastric cancer. Mol. Biol. Rep..

[67] Li B.K., Huang P.Z., Qiu J.L., Liao Y.D., Hong J., Yuan Y.F. (2014). Upregulation of microRNA-106b is associated with poor prognosis in hepatocellular carcinoma. Diagn. Pathol..

[68] Yang T.S., Yang X.H., Chen X., Wang X.D., Hua J., Zhou D.L., Zhou B., Song Z.S. (2014). MicroRNA-106b in cancer-associated fibroblasts from gastric cancer promotes cell migration and invasion by targeting PTEN. FEBS Lett..

[69] Gottmann P., Ouni M., Saussenthaler S., Roos J., Stirm L., Jahnert M., Kamitz A., Hallahan N., Jonas W., Fritsche A. (2018). A computational biology approach of a genome-wide screen connected miRNAs to obesity and type 2 diabetes. Mol. Metab..

[70] Marcuello M., Duran-Sanchon S., Moreno L., Lozano J.J., Bujanda L., Castells A., Gironella M. (2019). Analysis of A 6-Mirna Signature in Serum from Colorectal Cancer Screening Participants as Non-Invasive Biomarkers for Advanced Adenoma and Colorectal Cancer Detection. Cancers.

[71] Yuan C., Zhang Y., Tu W., Guo Y. (2019). Integrated miRNA profiling and bioinformatics analyses reveal upregulated miRNAs in gastric cancer. Oncol. Lett..

[72] Li C.Y., Zhang W.W., Xiang J.L., Wang X.H., Li J., Wang J.L. (2019). Identification of microRNAs as novel biomarkers for esophageal squamous cell carcinoma: A study based on The Cancer Genome Atlas (TCGA) and bioinformatics. Chin. Med. J..

[73] Lu L., Li Y., Wen H., Feng C. (2019). Overexpression of miR-15b Promotes Resistance to Sunitinib in Renal Cell Carcinoma. J. Cancer.

[74] Sun G., Shi L., Yan S., Wan Z., Jiang N., Fu L., Li M., Guo J. (2014). MiR-15b targets cyclin D1 to regulate proliferation and apoptosis in glioma cells. BioMed Res. Int..

[75] Zheng L., Qi T., Yang D., Qi M., Li D., Xiang X., Huang K., Tong Q. (2013). microRNA-9 suppresses the proliferation, invasion and metastasis of gastric cancer cells through targeting cyclin D1 and Ets1. PLoS ONE.

[76] Wang F., Ren X., Zhang X. (2015). Role of microRNA-150 in solid tumors. Oncol. Lett..

[77] Vasilatou D., Papageorgiou S., Pappa V., Papageorgiou E., Dervenoulas J. (2010). The role of microRNAs in normal and malignant hematopoiesis. Eur. J. Haematol..

[78] Jiang X., Huang H., Li Z., Li Y., Wang X., Gurbuxani S., Chen P., He C., You D., Zhang S. (2012). Blockade of miR-150 maturation by MLL-fusion/MYC/LIN-28 is required for MLL-associated leukemia. Cancer Cell.

[79] Watanabe A., Tagawa H., Yamashita J., Teshima K., Nara M., Iwamoto K., Kume M., Kameoka Y., Takahashi N., Nakagawa T. (2011). The role of microRNA-150 as a tumor suppressor in malignant lymphoma. Leukemia.

[80] Fulci V., Chiaretti S., Goldoni M., Azzalin G., Carucci N., Tavolaro S., Castellano L., Magrelli A., Citarella F., Messina M. (2007). Quantitative technologies establish a novel microRNA profile of chronic lymphocytic leukemia. Blood.

[81] Agirre X., Jimenez-Velasco A., San Jose-Eneriz E., Garate L., Bandres E., Cordeu L., Aparicio O., Saez B., Navarro G., Vilas-Zornoza A. (2008). Down-regulation of hsa-miR-10a in chronic myeloid leukemia CD34+ cells increases USF2-mediated cell growth. Mol. Cancer Res. MCR.

[82] Zanette D.L., Rivadavia F., Molfetta G.A., Barbuzano F.G., Proto-Siqueira R., Silva W.A., Falcao R.P., Zago M.A. (2007). miRNA expression profiles in chronic lymphocytic and acute lymphocytic leukemia. Braz. J. Med. Biol. Res. = Rev. Bras. Pesqui. Med. E Biol..

[83] Xu L., Liang Y.N., Luo X.Q., Liu X.D., Guo H.X. (2011). Association of miRNAs expression profiles with prognosis and relapse in childhood acute lymphoblastic leukemia. Zhonghua Xue Ye Xue Za Zhi = Zhonghua Xueyexue Zazhi.

[84] Bender T.P., Kremer C.S., Kraus M., Buch T., Rajewsky K. (2004). Critical functions for c-Myb at three checkpoints during thymocyte development. Nat. Immunol..

[85] Wang X., Angelis N., Thein S.L. (2018). MYB—A regulatory factor in hematopoiesis. Gene.

[86] Nicot C., Mahieux R., Pise-Masison C., Brady J., Gessain A., Yamaoka S., Franchini G. (2001). Human T-cell lymphotropic virus type 1 Tax represses c-Myb-dependent transcription through activation of the NF-kappaB pathway and modulation of coactivator usage. Mol. Cell. Biol..

[87] Nakano K., Uchimaru K., Utsunomiya A., Yamaguchi K., Watanabe T. (2016). Dysregulation of c-Myb Pathway by Aberrant Expression of Proto-oncogene MYB Provides the Basis for Malignancy in Adult T-cell Leukemia/lymphoma Cells. Clin. Cancer Res. Off. J. Am. Assoc. Cancer Res..

[88] Chang T.C., Yu D., Lee Y.S., Wentzel E.A., Arking D.E., West K.M., Dang C.V., Thomas-Tikhonenko A., Mendell J.T. (2008). Widespread microRNA repression by Myc contributes to tumorigenesis. Nat. Genet..

[89] Chang T.C., Zeitels L.R., Hwang H.W., Chivukula R.R., Wentzel E.A., Dews M., Jung J., Gao P., Dang C.V., Beer M.A. (2009). Lin-28B transactivation is necessary for Myc-mediated let-7 repression and proliferation. Proc. Natl. Acad. Sci. USA.

[90] D’Agostino D.M., Raimondi V., Silic-Benussi M., Ciminale V. (2022). MiR-150 in HTLV-1 infection and T-cell transformation. Front. Immunol..

[91] Al Sharif S., Pinto D.O., Mensah G.A., Dehbandi F., Khatkar P., Kim Y., Branscome H., Kashanchi F. (2020). Extracellular Vesicles in HTLV-1 Communication: The Story of an Invisible Messenger. Viruses.

[92] Wang X., Tang S., Le S.Y., Lu R., Rader J.S., Meyers C., Zheng Z.M. (2008). Aberrant expression of oncogenic and tumor-suppressive microRNAs in cervical cancer is required for cancer cell growth. PLoS ONE.

[93] Motsch N., Pfuhl T., Mrazek J., Barth S., Grasser F.A. (2007). Epstein-Barr virus-encoded latent membrane protein 1 (LMP1) induces the expression of the cellular microRNA miR-146a. RNA Biol..

[94] Jazdzewski K., Murray E.L., Franssila K., Jarzab B., Schoenberg D.R., de la Chapelle A. (2008). Common SNP in pre-miR-146a decreases mature miR expression and predisposes to papillary thyroid carcinoma. Proc. Natl. Acad. Sci. USA.

[95] Lin S.L., Chiang A., Chang D., Ying S.Y. (2008). Loss of mir-146a function in hormone-refractory prostate cancer. RNA.

[96] Nahand J.S., Karimzadeh M.R., Nezamnia M., Fatemipour M., Khatami A., Jamshidi S., Moghoofei M., Taghizadieh M., Hajighadimi S., Shafiee A. (2020). The role of miR-146a in viral infection. IUBMB Life.

[97] Tomita M., Tanaka Y., Mori N. (2012). MicroRNA miR-146a is induced by HTLV-1 tax and increases the growth of HTLV-1-infected T-cells. Int. J. Cancer.

[98] Hachiman M., Yoshimitsu M., Ezinne C., Kuroki A., Kozako T., Arima N. (2018). In vitro effects of arsenic trioxide, interferon alpha and zidovudine in adult T cell leukemia/lymphoma cells. Oncol. Lett..

