# Supplementary material for: Small RNA Profiling in an HTLV-1-Infected Patient with Acute Adult T-Cell Leukemia-Lymphoma at Diagnosis and after Maintenance Therapy: A Case Study
Source: Int J Mol Sci. 2023 Jun 26;24(13):10643. doi: 10.3390/ijms241310643 (PMC10342019; doi:10.3390/ijms241310643)
Supplement: Supplementary file 1 [file ijms-24-10643-s001.zip › Supplementary Figure 1/Legend to supplementary figure 1.pdf]

Legend to supplementary figure S1

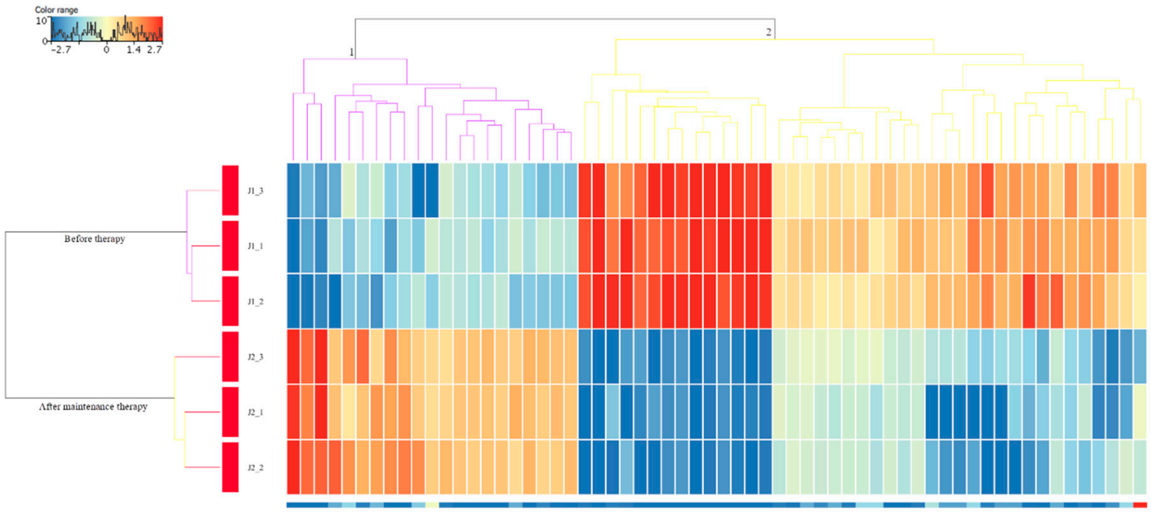

- **Figure S1**. Heatmap showing the results of a hierarchical cluster analysis for the two samples groups and the 62 significantly dysregulated novel sRNAs. The sample cluster tree is shown on the left, with the sRNA cluster tree on top. The color scale at the top indicates the relative expression level of sRNA in all samples. Red means that the expression levels are higher than the mean, while blue means that the expression levels are lower than the mean. Each column represents a novel sRNA, and each row represents a sample.
